# Impact of the Frequency and Type of Procedures Performed in Nuclear Medicine Units on the Expected Radiological Hazard

**DOI:** 10.3390/ijerph20065206

**Published:** 2023-03-15

**Authors:** Katarzyna Matusiak, Justyna Wolna, Aleksandra Jung, Leszek Sadowski, Jolanta Pawlus

**Affiliations:** 1 Faculty of Physics and Applied Computer Science, AGH University of Science and Technology, Av. Mickiewicza 30, 30-059 Krakow, Poland; 2 Department of Nuclear Medicine, 5th Military Hospital, ul. Wroclawska 1-3, 30-901 Krakow, Poland

**Keywords:** TLD, dose estimation, nuclear medicine procedures, medical staff radiological exposure

## Abstract

Nuclear medicine procedures play an important role in medical diagnostics and therapy. They are related to the use of ionizing radiation, which affects the radiological exposure of all of the persons involved in their performance. The goal of the study was to estimate the doses associated with the performance of various nuclear medicine procedures in order to optimize workload management. The analysis was performed for 158 myocardial perfusion scintigraphy procedures, 24 bone scintigraphies, 9 thyroid scintigraphies (6 with use of ^131^I and 3 with ^99m^Tc), 5 parathyroid glands and 5 renal scintigraphies. In this evaluation, two possible locations of thermoluminescent detectors, used for measurements, were taken into consideration: in the control room and directly next to the patient. It was shown how the radiological exposure varies depending on the performed procedure. For high activity procedures, ambient dose equivalent registered in the control room reached the level over 50% of allowed dose limit. For example, ambient dose equivalent obtained in control room when performing bone scintigraphy only was 1.13 ± 0.3 mSv. It is 68% of calculated dose limit in the examined time span. It has been shown that risk associated with nuclear medicine procedures is influenced not only by the type of procedure, but also by the frequency of their performance and compliance with the ALARA principle. Myocardial perfusion scintigraphy accounted for 79% of all evaluated procedures. The use of radiation shielding reduced the obtained doses from 14.7 ± 2.1 mSv in patient’s vicinity to 1.47 ± 0.6 mSv behind the shielding. By comparing the results obtained for procedures and dose limits established by Polish Ministry of Health, it is possible to estimate what should be the optimal division of duties between staff, so that everyone receives similar doses.

## 1. Introduction

Examinations of organ functions and structures or diseases treatments based on open radioactive sources are increasingly common [1]. According to the reports of The Society of Nuclear Medicine & Nuclear Imaging, there are about 20 million nuclear medicine procedures performed every year in the United States [2]. After radioactive isotope administration, the patient becomes an “open source of radiation”, which means higher radiological exposure to medical employees in nuclear medicine (NM) departments compared to workers in radiological units.

To minimize consequences of radiation detriment, the As Low As Reasonably Achievable (ALARA) rule is applied [3]. According to this rule all, of administered doses should be “as low as reasonably achievable”, taking into account economic and social factors [3,4]. It is known that over-reducing of the dose may have adverse effects on diagnostic information, while the over-increasing of the dose level may increase the radiological hazard of the patient and medical staff. Therefore, it is very important to adjust injected radioactivity to given conditions while ensuring the maximum protection of personnel involved in the diagnostics process [5].

Due to the fact that it is impossible to completely avoid exposure while working in NM department [6,7] acceptable effective doses for medical personnel have been established. These doses are presented in International Commission on Radiation Protection (ICRP) reports [8], in the ordinances of the Ministry of Health, in the Atomic Law [9,10] and the European Union Council Directive 96/29/EUROATOM [11]. The above-mentioned acts also specify whether dosimetric measurements should be carried out individually for each employee or in the work-environment. In order to adapt the method of dosimetric measurements, two categories of employees were introduced: category A and B. The employee of category A is one who may be exposed to an effective dose higher than 6 mSv/year. This category is subject to individual dosimetry. Employees who are at risk of exposure below 6 mSv/year are employees of category B and are under general dosimetric surveillance [9,10,11]. Most of NM medical staff fall into category B.

Estimation of radiological exposure of both patients and medical staff is the main subject of many publications [12,13,14,15,16,17,18]. Most of those related to the staff radiation exposure estimation describe research conducted from the perspective of radiological protection of a single worker [6,7,19]. Individual doses were measured using different detectors, e.g., TLD or electronic pocket dosimeters (EPD). In the case of PET scans, the average dose received by the technician for a single treatment is approximately 10 nSv/MBq of the administered activity [7]. The situation for SPECT procedures is similar and obtained doses are approximately equal 1–3 μSv/GBq of the administered radioactivity [19]. In both mentioned cases, it is not possible to state unequivocally which procedure is associated with potentially the highest dose because the value of the activity administered to the patient might not have to correlate directly with the external radiological risk, due to the different kinetics of the administered radiopharmaceuticals. Therefore, a study aimed at associating the radiation hazard with the type of procedure performed is advisable. It is expected that longer observation is more adequate as the dose assessment for a single procedure would be related to too much high uncertainty related to the detection limit.

This approach allows for the estimation of the dose per so-called virtual individual worker, for which we can assume the type and number of performed procedures. Meanwhile, determination of the dose related to the procedure may allow for optimization of work division work in NM department. Therefore, the main goal of this study was to identify the radiological exposure associated with a chosen procedures by calculating the average ambient equivalent dose per procedure in the direct vicinity of the patient and in the control room.

## 2. Materials and Methods

### 2.1. Thermoluminescent Detectors (TLD)

In order to estimate radiological exposure, both in the control room and next to the patient, it was necessary to use detectors which would not interfere with nuclear imaging procedure but would also allow for an accurate estimation of exposure. Considering their small size and wide measuring range, TLD meet these requirements.

In this study, 35 MCP-N (LiF:Mg,Cu,P) thermoluminescent detectors produced by the Institute of Nuclear Physics PAN in Krakow were used. They were divided into 12 groups and put in transparent plastic bags. Six groups were used for dose measurements near the patient, while the other six were intended for measurements in the control room.

Detectors designated for measurements next to the patient were placed on the gantry of apparatus, which was as close as possible to the patient while the study was proceeding. Groups of detectors intended for measurements in the control room were placed on the desk, next to the control panel of the gamma camera. Detectors were used for measurements only when the dedicated type of the procedure was performed. Otherwise, they were stored in a lead container to prevent accidental contamination.

**Figure 1:**
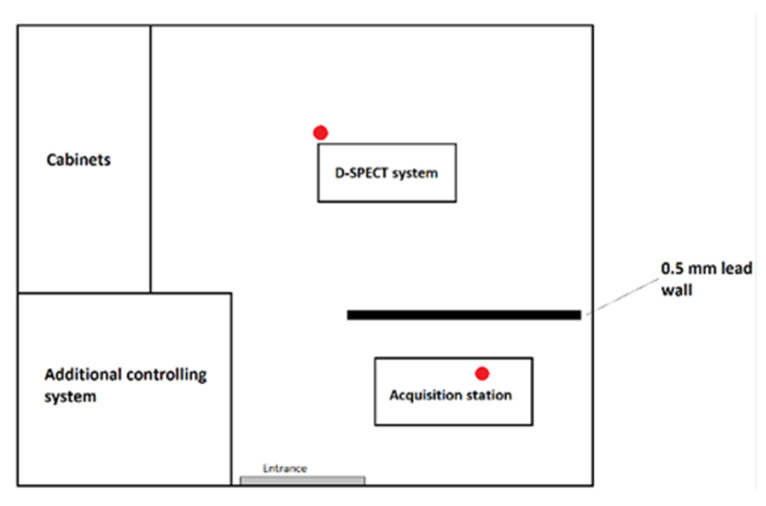
Schematic drawing of D-SPECT acquisition room with detectors’ locations (red circles).

**Figure 2:**
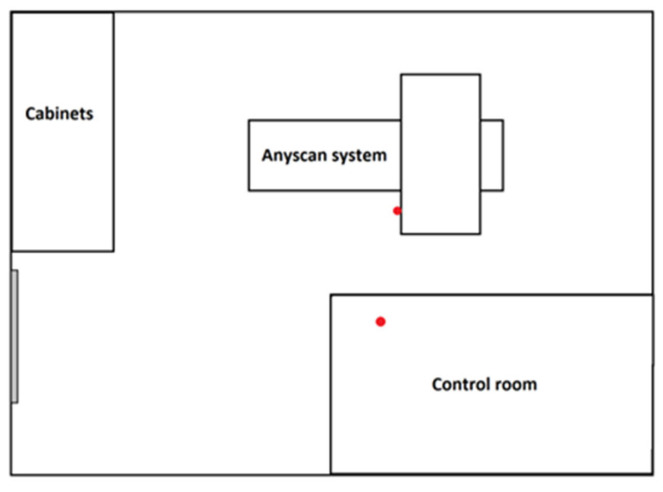
Schematic drawing of Mediso Anyscan acquisition room with detectors’ locations (red circles).

**Figure 3:**
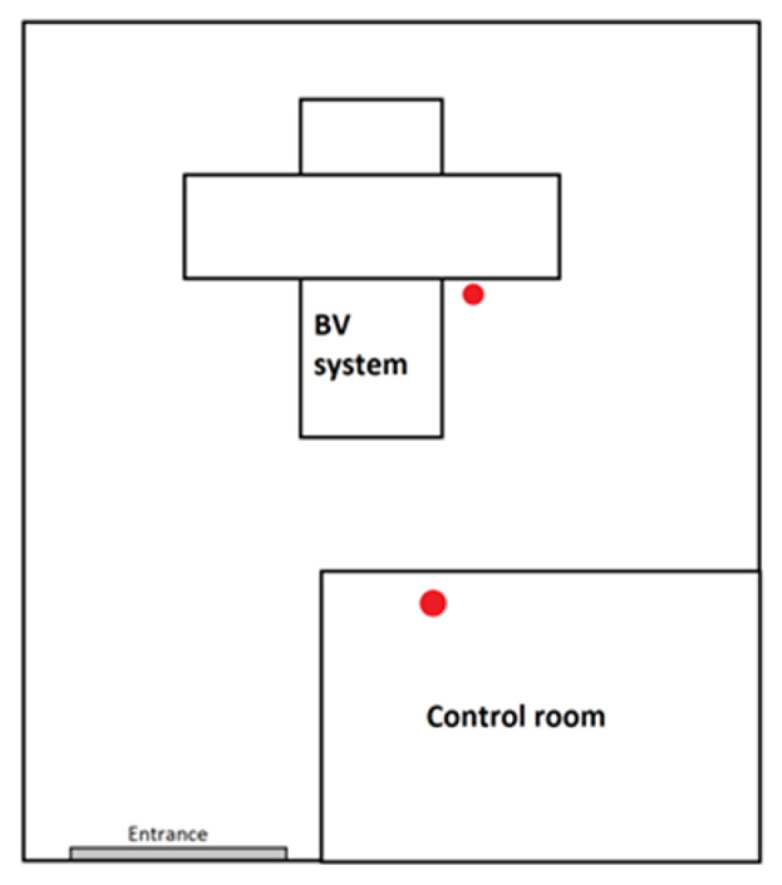
Schematic drawing of Philips BrightView acquisition room with detectors’ locations (red circles).

In Figure 1, Figure 2 and Figure 3, schematic drawings of acquisition rooms with location of the detectors during the measurements (marked as red full circles) are presented. The D-SPECT gamma camera, produced by Spectrum Dynamics Medical, was dedicated to myocardial perfusion scintigraphy only due to its unique open gantry design. Mediso AnyScan and Philips BrightView gamma cameras were used interchangeably for the remaining procedures, as these are regular devices with circular gantry.

The calculations presented in this article were based on scintigraphic procedures performed in one of the departments of nuclear medicine in Krakow. Due to the strong interest in determining the dose related to myocardial perfusion scintigraphy, data acquisition was completed in 2 months (for better accuracy) and for the remaining procedures in 1 month, mostly for comparison purposes.

Before the main measurements, TLDs were properly prepared. Individual response factors (IRF) and calibration curve were determined. In both of the procedures, gamma ray ^137^Cs calibration source was used (source activity on the day of irradiation was equal to 0.14 GBq). TLDs were irradiated in the same geometry to obtain repeatable doses. Before and after irradiation, all detectors underwent pre- and post-irradiation annealing in TLDO oven (240 °C for 10 min and 100 °C for 10 min, respectively), produced by PTW, followed by rapid cooling down [20,21,22]. The readout process was carried out using TL manual reader RA’04, produced by MICROLAB, in the three-step plateau heating mode: 150 °C for 10 s, 245 °C for 15 s and, finally, 245 °C for 10 s. The photomultiplier window was open only in the second stage of heating and counts were then registered. Individual response factors were calculated in order to compensate differences in the sensitivity of the used detectors [20,23]. They were calculated as the response of the individual count in the detector to the average counts of the whole dosimeters batch for the first irradiation to the same dose. In addition, in order to eliminate research detectors with significantly different IRF, 3 Sigma rule was applied to increase the repeatability of measurements. Only detectors with IRF values within the average value in the range of IRF ± 3ϭ were allowed for further measurements. In the presented case, the average IRF value was equal to 1.02 ± 0.14 and the ϭ value was calculated as the estimator of the standard deviation. All of the IRF values were within the given range; therefore, none of the detectors were rejected from further analysis. In addition, obtained results for single TLD were corrected by the corresponding IRF.

The information obtained from the readout of thermoluminescent detectors is not an absolute dose value. In order to calculate the doses absorbed by the detectors, it was necessary to determine the calibration curve by irradiating detectors with the defined doses ranging from 0.17 mGy to 1 mGy and fitting the calibration curve (described by linear equation) to given points. The linear dose-response characteristics of TLD was confirmed by earlier works [24,25]. Equation (1) stands for calibration equation and was later on used to calculate doses acquired during measurements.

(1) n=D+252

where:

*D*—dose absorbed by the detectors [mGy];

*n*—thermolumienscent signal expressed as counts number.

The relative uncertainty of *a* and *b* parameters was, respectively, 5.2% and 4.8%.

Combining IRF numbers and the calibration Equation (1), the absorbed dose was calculated as:

(2) $$D=\frac{IRF\cdot n-252}{1011}$$

The last step of TLDs preparation procedure was the pre-exposure annealing at the same temperature and timing as for the calibration procedure.

After exposure at the Department of Nuclear Medicine, the readout procedure was carried at temperatures described below, preceded by the post-exposure annealing.

### 2.2. Equipment

In the presented work, the distribution of the radiotracer in patient’s body was imaged using three different gamma cameras:

1. double-head gamma camera AnyScan SC SPECT/CT, produced by Mediso;
2. double-head gamma camera BrightView XCT SPECT/CT, produced by Philips;
3. cardiac CZT gamma camera D-SPECT, produced by Spectrum Dynamics Medical.

### 2.3. Selected Nuclear Medicine Procedures

The following procedures were analyzed in the presented study.

1. Myocardial perfusion scintigraphy—the reference activity was 800 MBq per injection but due to using CZT gamma camera, while performing the presented study, it was possible to reduce injected radioactivity by 50% in relation to the reference value [26]. Study duration: 5–12 min [27]. Data obtained from 158 patients (105 women, 53 men) were taken into account. In this group, 76 rest and 82 stress myocardial perfusion scintigraphy procedures were performed. The average administered radioactivity was 396 ± 74 MBq.
2. Bone scintigraphy—the reference activity was 740 MBq. Study duration of the delayed phase: 20 min [27]. The analyzed data consist of 24 patients (14 women and 10 men). The average administered radioactivity was 722 ± 103 MBq.
3. Renal scintigraphy—in the case of the presented work performed in dynamic protocol. The reference radioactivity ranged from 70 to 200 MBq. Study duration: 20–30 min [27]. Data obtained from nine patients (six with use of ^131^I and three with ^99m^Tc) were taken into account.
4. Parathyroid glands scintigraphy—acquisition was carried out with two different radiotracers: ^99m^TcO_4_ and ^99m^Tc-MIBI. The reference radioactivity for thyroid study was 80 MBq for the test with ^99m^TcO_4_ and 500–740 MBq for imaging with ^99m^Tc-MIBI. For imaging with ^99m^Tc-MIBI, imaging was performed twice—20 min and 120 min after injection. Duration of each part of the study: 15 min [27]. The analyzed data consist of five patients.
5. Thyroid scintigraphy—typically performed using two isotopes: ^131^I or ^99m^Tc which depended on clinical indications. In the case of ^131^I the reference, the activity was 7.4 MBq, while for ^99m^Tc, it was 80 MBq. Study duration for each studies: 15–30 min [27]. The analyzed data consist of five patients.

In all of the presented cases, the reference injected radioactivity depended on patients weight, age and sex. Conversion factors were used to determine the dose for each patient [27]. It was also justified to increase or decrease administered radioactivity under specific clinical conditions, e.g., test performed on emaciated patient (decreased radioactivity) or suffering patient, who will not be able to remain still for a long time (increased radioactivity). The differences from the reference radioactivity values and time of measurement were included in the average dose calculation for each procedure.

## 3. Results

The absorbed doses during the subsequent procedures were determined using thermoluminescent detectors. Then, the obtained values were calculated to the ambient dose equivalent H*(10) [12,13] and compared with the dose limits established by the ICRP, the polish Atomic Law and the EUROATOM Directive.

### 3.1. Absorbed Doses

Based on computed calibration equation (Equation (2)), it was possible to estimate the doses absorbed by the detectors, which are presented in Table 1. The uncertainty of the doses was calculated from the propagation of the uncertainty law.

**Figure 4:**
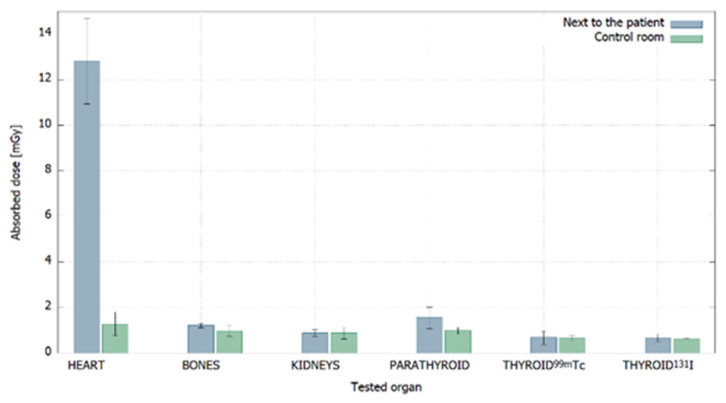
Comparison of the doses absorbed by TLDs located next to the patient and in the control room for all procedures.

In Figure 4, the doses absorbed by the TL detectors are shown.

### 3.2. Comparison with Dose Limits

The next stage of the data analysis was the comparison of the absorbed doses with the dose limits established by the ICRP, [8] the Polish Ministry of Health [9,10] and the directive EUROATOM [11]. In general, for medical staff, the dose limit is 20 mSv/year. In the presented study, the acquisition time was 2 months for myocardial perfusion scintigraphy and 1 month for other procedures. The calculated dose limits based on the above-mentioned regulations and different observation time were: 1.67 mSv/month for all procedures except for myocardial perfusion scintigraphy and 3.33 mSv/2 months for myocardial perfusion scintigraphy. By comparing the results obtained for procedures and annual limits, it is possible to estimate what should be the optimal division of duties between staff so that everyone receives similar individual dose

In order to compare the results with the dose limits, it was necessary to make conversions from the absorbed dose [mGy] to the ambient dose equivalent [mSv] (Table 2).

In total, 201 scintigraphic procedures were performed. In Table 3, information about how many times each procedure was performed, the share of each procedure in total number of performed tests (expressed as percentage of total number of procedures) and calculated H*(10) per single procedure of each type is shown.

The dosimetric measurements in this study were taken not individually, for each employee, but through general dosimetric surveillance. This is why, in order to properly estimate radiological hazard, it was necessary to assume the time spent in both of the locations. It was estimated that the technician spends an average of 20% of the procedure’s time in the vicinity of the patient, positioning him, and 80% of the time in the control room. The results of these calculations are presented in Table 3.

The data presented in Table 3 are a kind of soft guideline for people managing the division of duties in a given nuclear medicine facility. Due to a different frequency of conducting various scintigraphic studies, these data will differ among NM departments. The data shown in this table contain numerical information on how often the given procedure is performed and what the approximate radiation exposure is. Based on this type of data and planned schedule of scintigraphic procedures, work can be divided in such a way that the frequency of performing high-activity procedures is similar for all technicians so none of them is exposed to a significantly larger dose than other employees.

## 4. Discussion

Many publications devoted to the assessment of occupational exposure focus on determining individual dose obtained by a single worker in relation to established dose limits [6,7,25,28]. The main purpose of this study was to provide quantitative data on ionizing radiation doses associated with selected nuclear medicine procedures and provide an additional method of minimizing radiological hazard than those mentioned in existing literature [6,7,29]. Namely, indicating possible strategies regarding how the personnel exposure might be equalized.

Measurements were performed for various scintigraphic procedures: myocardial perfusion scintigraphy, bone scintigraphy, thyroid scintigraphy, parathyroid glands scintigraphy and renal scintigraphy. Two possible locations of the detectors were taken into account in the calculations: the spot next to the gantry of apparatus (ergo directly next to the patients) and the control room, behind radiation shields. It is obvious that while performing procedure, the technician will not spend the whole time next to the patient, nor in the control room. There is some time needed for positioning the patient; however, after this part of the procedure is done, medical employees stay as far as possible in a shielded space. The purpose of this publication was to estimate the maximum doses for the procedure; therefore, it was decided to use area monitoring rather than individual dosimetry. Some patients require more effort while positioning them, and some even the constant presence of a guardian. Measurements carried out in the control room are supposed to represent the most optimistic situation, in which the personnel limits contact with the patient only to necessary interactions. However, this is not always possible.

In this study, thermoluminescent detectors were used. These detectors are often used in medical dosimetry mainly due to their small size, which allows dose measurements without disturbing patients or medical staff [30].

Basing on the equation of calibration’s curve, it was possible to determine the unknown doses absorbed by TLD, which are presented in Table 1 and Figure 4. It is apparent that the highest dose was absorbed by detectors intended for measurements during myocardial perfusion scintigraphy. The detectors intended for measurements next to the patient absorbed a dose of 12.8 ± 1.8 mGy, while the detectors located in the control room absorbed a dose of 1.28 ± 0.5 mGy. This may be explained by the longest time of acquisition (2 months), the largest number of performed procedures (158) and the characteristic of the radiotracer.

The global analysis of Table 1 indicated that for high activity procedures such as myocardial perfusion scintigraphy, bone scintigraphy or parathyroid glands scintigraphy, the doses absorbed by the detectors located next to the patient were, as expected, higher than the doses absorbed by the detectors placed in the control room. This means that shielding designed for examination rooms and distance from patients were enough to provide radiological protection for the personnel. For low-activity procedures, such as renal scintigraphy or thyroid scintigraphy, the doses absorbed next to the patient and in the control room are comparable. Such a result could indicate a serious problem with adequate shielding; however, it should be remembered that the same shielding was effective for high-activity procedures. The lack of a significant difference may indicate that the number of evaluated procedures was too low to obtain reliable results. This is a rationale for further research on this topic, with a particular focus on low-activity procedures.

Another reason for this result could be that, in the case of low-activity procedures, the absorbed doses mainly come from the radiological background in the acquisition room. It is noticeable that obtained results for renal, ^131^I and ^99m^Tc thyroid scintigraphies are slightly different (result from control room 0.88 ± 0.25 mGy, 0.64 ± 0.03 mGy and 0.65 ± 0.12 mGy, respectively). This may be influenced by the fact that in the acquisition rooms presented in Figure 2 and Figure 3, high activity procedures, such as bone or parathyroid glands scintigraphy, were also performed. If right after performing such a procedure lower activity one was carried on, the momentary radiological background in the area could have been raised. This tendency is particularly important in the presented case, due to the small number of studied procedures; therefore, average doses are especially susceptible to background fluctuations.

The final part of the analysis of the results was comparison of the obtained data with dose limits for medical staff, which is 20 mSv/year. This comparison is presented in Table 2. It is clear that the difference in H*(10) values for detectors located close to the patient and for detectors located in the control room is much more significant for high activity procedures. The data included in Table 2 also indicate that, in case of myocardial perfusion or parathyroid glands scintigraphy, the estimated doses carry a risk of exceeding the dose limit. Such a situation may occur when the patient’s positioning takes much longer than expected. The doses obtained in the control room are within the acceptable limits; however, their values are also increased, which means that in case of a significant number of performed procedures, an appropriate division of duties among the employees would be recommended.

According to the literature, the number of performed nuclear medicine diagnostic procedures has increased over the years. Therefore, special attention should be paid to methods and regulations allowing for better control of occupational doses [31].

In the presented study, although the highest total dose was obtained for myocardial perfusion scintigraphy, the highest dose per procedure was for parathyroid glands scintigraphy (Table 3). Therefore, when planning the examination schedule, care should be taken to ensure that technicians performing parathyroid glands scintigraphy do not perform myocardial scintigraphy or perform it in a limited extent due to increased levels of injected radioactivity. In this way, disproportion in the doses received by the employees that could occur can be prevented [19].

Staying away from the patient during the procedure significantly reduces the expected radiological exposure. In all of the presented cases, the H*(10) value estimated for the detectors located in the control room is below the established dose limits (Table 2). However, it is important to remember that in clinical practice, technicians need to spend some time in close proximity to the patient, e.g., before scanning, while positioning them on the table. For this reason, an approximation of the time spent in two locations was carried out. The time division is described in Section 3.2. *A Comparison with dose limit* and results of these calculations are presented in Table 3. There was a slight increase in values after time correction in comparison with doses in control room only for myocardial perfusion scintigraphy and for parathyroid glands scintigraphy. The differences for the remaining procedures were below the threshold of accuracy adopted in the presentation of the results. This result may also be caused by the small number of studied procedures. Nonetheless, it should be assumed that even if the ALARA rule is followed, the dose values may be higher than those presented in this article; thus, the limits for category B employees may be exceeded. It is worth remembering, especially when only area dosimetry is carried out.

## 5. Conclusions

Most publications on the assessment of occupational exposure and radiological hazard in nuclear medicine departments focus on obtaining individual doses for each employee. Meanwhile, the main scope of this publication focuses on indicating the maximum dose received during different procedures. This information can be an indicator for optimization of duties, leading to lowering and equalizing doses acquired by medical personnel.

It is important to remember that radiological exposure is related to the type of performed procedure, not only because of the administered activity, but also because of the frequency of its performance and possibility of difficulties in the proper positioning of the patient. In clinical work, not every diagnostic procedure is performed without obstacles. Therefore, estimating maximum doses improves workers safety and follows the principle of pessimization. Although results presented in this study may be ambiguous, they provide a basis for continuing studies of this type in an expanded scope. This publication was focused solely on scintigraphic procedures, while PET/CT procedures are a major contribution to nuclear medicine. According to the literature, workers performing this type of procedure are more exposed to ionizing radiation than those performing scintigraphic procedures [32]. Thus, the calculations presented in this publication may provide a basis for improving work schedules for PET/CT employees as well.

## Figures and Tables

**Table 1:** The total doses absorbed at the point of interest for two- or one-month observation periods through TLD with their uncertainties.

Assignment	Average Counts from Group	Dose [mGy]
**Near the Patient **		
• Heart	13,211 ± 1900	12.8 ± 1.8
• Bones	1475 ± 97	1.21 ± 0.10
• Kidneys	1150 ± 159	0.89 ± 0.16
• Parathyroid glands	1816 ± 472	1.55 ± 0.47
• Thyroid (^99m^Tc)	941 ± 306	0.68 ± 0.30
• Thyroid (^131^I)	927 ± 164	0.67 ± 0.16
**In the Control Room **		
• Heart	1541 ± 521	1.27 ± 0.52
• Bones	1241 ±261	0.98 ± 0.26
• Kidneys	1139 ± 252	0.88 ± 0.25
• Parathyroid glands	1250 ± 152	0.99 ± 0.15
• Thyroid (^99m^Tc)	907 ± 123	0.65 ± 0.12
• Thyroid (^131^I)	895 ± 30	0.64 ± 0.03

**Table 2:** Ambient dose equivalent expressed as percentage of the dose limit.

**Near the Patient **			
**Dose Limit **	**Tested Organ **	H*(10) [mSv]	**Percentage of Dose Limit **
1.67 mSv/month	Bone	1.39 ± 0.11	83%
	Kidneys	1.02 ± 0.17	61%
	Thyroid ^99m^Tc	0.78 ± 0.34	47%
	Thyroid ^131^I	0.77 ± 0.18	46%
	Parathyroid glands	1.78 ± 0.52	101%
3.33 mSv/2 months	Heart	14.7 ± 2.1	442%
**In the Control Room **			
**Dose Limit **	**Tested Organ **	H*(10) [mSv]	**Percentage of Dose Limit **
1.67 mSv/month	Bone	1.13 ± 0.30	68%
	Kidneys	1.01 ± 0.29	61%
	Thyroid ^99m^Tc	0.75 ± 0.13	45%
	Thyroid ^131^I	0.74 ± 0.03	44%
	Parathyroid glands	1.14 ± 0.17	68%
3.33 mSv/2months	Heart	1.47 ± 0.6	44%

**Table 3:** Share in the total number of procedures, calculated ambient equivalent doses for a single study and ambient equivalent dose for a study including time spent in each of the locations.

			Near the Patient	In Control Room			
Tested Organ	Number of Procedures	Share in Total Number of Procedures	H*(10)/Study [mSv]	H*(10) Time Correction [mSv]	H*(10)/Study [mSv]	H*(10) Time Correction [mSv]	Total Dose [mSv]
Heart	158	79%	0.09	0.02	0.01	0.01	0.03
Bones	24	12%	0.06	0.01	0.05	0.04	0.05
Kidneys	5	2%	0.20	0.04	0.20	0.16	0.20
^131^I Thyroid	6	3%	0.13	0.03	0.12	0.10	0.12
^99m^Tc Thyroid	3	1%	0.26	0.05	0.25	0.20	0.25
Parathyroid glands	5	2%	0.36	0.07	0.23	0.18	0.26

## Data Availability

Not applicable.
